# Comparison of arthroscopic debridement and microfracture in the treatment of osteochondral lesion of talus

**DOI:** 10.3389/fsurg.2022.1072586

**Published:** 2023-01-13

**Authors:** Minghua Zhang, Daohua Chen, Qiang Wang, Ying Li, Shiming Huang, Peng Zhan, Jiajing Lai, Jianqing Jiang, Dongfeng Chen

**Affiliations:** Department of Bone and Joint Sports Medicine, Longyan First Affiliated Hospital of Fujian Medical University, Longyan, China

**Keywords:** osteochondral lesion of talus, ankle arthroscopy, debridement, microfracture, BMI

## Abstract

**Objective:**

This study was performed to compare the clinical effect of arthroscopic debridement vs. arthroscopic microfracture in the treatment of osteochondral lesions of the talus.

**Methods:**

We retrospectively reviewed patients with osteochondral lesion of talus who were admitted to our hospital from April 2020 to April 2021. The patients were divided into Group A (arthroscopic debridement group, *n* = 39) and Group B (arthroscopic microfracture group, *n* = 42), and the intraoperative details in the two groups were analyzed. The American Orthopaedic Foot and Ankle Society (AOFAS) score and visual analogue scale (VAS) score were compared between the two groups before surgery and at the last follow-up.

**Results:**

The postoperative AOFAS score (Group A, 40.9–82.26; Group B, 38.12–87.38), VAS score (Group A, 6.44–3.92; Group B, 6.38–2.05) significantly improved in both groups, but the improvement was significantly greater in Group B than in Group A (*P *< 0.05). Among all patients, the AOFAS and VAS scores of men aged ≤30 years and patients with a low body mass index (BMI) improved more significantly (*P *< 0.05).

**Conclusion:**

The arthroscopic microfracture for the treatment of osteochondral lesion of talus is superior to joint debridement in terms of improving ankle function, especially in relatively young men with a relatively low BMI.

## Introduction

Ankle sprain is the most common injury in sports (1). Osteochondral lesions of the talus (OLTs) are particularly common; such lesions involve any impairment of the articular surface of the talus or subchondral bone (2). These defects often cause symptoms such as deep ankle pain, swelling, weakness, a locking sensation, and instability at the ankle (3–5). The risk factors and etiology of OLTs include acute and severe ankle sprain, fracture, and recurrent ankle sprain. Nontraumatic causes include local osteonecrosis, systemic vascular disease, and congenital or endocrine abnormalities (3). However, because the articular cartilage shows limited healing and regeneration abilities (6), spontaneous healing of OLTs to normal cartilage rarely occurs (7, 8). Recent studies have shown that conservative management of OLTs has poorer outcomes than operative treatment (9, 10).

Various operative treatments of symptomatic OLTs have been reported, such as microfracture, debridement, osteochondral autograft transplantation, subchondral drilling, and autologous chondrocyte implantation (4). Debridement, microfracture, and subchondral drilling are performed for primary lesions of <1.5 cm in diameter (11, 12). These techniques are commonly performed arthroscopically using curettes and an arthroscopic shaver to remove surrounding unstable cartilage. For lesions of <10 mm in diameter, better results may be obtained by debridement combined with microfracture (13, 14). Osteochondral autograft transplantation is suitable for the treatment of total articular cartilage damage with or without subchondral bone cysts (15). Autologous chondrocyte implantation is performed in patients with large cartilage surface lesions in the talus (16). Among these techniques, arthroscopic microfracture and debridement are effective treatments for OLTs. Debridement creates a stable bleeding base through cleanup and curettage (17), and microfracture facilitates cartilage regeneration through bone marrow stimulation. These treatments have the advantages of a simple operation, minimal trauma, high safety and specificity, low cost, and mild postoperative pain (18, 19). However, whether articular debridement or microfracture is the best treatment for osteochondral lesion of talus remains controversial (5, 20). The effect of body mass index (BMI) on the outcome of arthroscopic treatment of OLTs remains unexplored. The present study was performed to compare the effect of ankle arthroscopic joint debridement vs. microfracture in the treatment of osteochondral lesion of talus, and differences in BMI were analyzed for clinical reference.

## Materials and methods

### General information

This study involved 105 patients with OLTs admitted to our hospital from April 2020 to April 2021. The inclusion criteria were an age of 18–<70 years; clinical manifestations such as ankle pain, swelling, stiffness, ankle instability, and tenderness in the injured area (2, 6, 13); imaging results that met the clinical diagnostic criteria for stage I and II OLTs; performance of ankle arthroscopic joint debridement or microfracture during hospitalization; and provision of written informed consent for participation in the study. There was no restriction on sex. The exclusion criteria were severe fractures or stiffness in other parts of the body and an osteochondral lesion area of >1.5 cm^2^. The discontinuation criteria were not following the doctor's advice during rehabilitation and the development of postoperative limb trauma or serious comorbidities.

Finally, after applying these criteria, 81 patients were included in the study. The patients were divided into Group A [arthroscopic debridement group, *n* = 39 (48.15%)] and Group B [arthroscopic microfracture group, *n* = 42 (51.85%)]. All patients had a single lesion. Group A comprised 26 men and 13 women with a mean age of 33.1 ± 11.86 years and cartilage injury area of 0.5–1.5 cm^2^ (mean, 1.01 ± 0.31 cm^2^). According to the magnetic resonance imaging (MRI) staging criteria (21), 6 patients had stage I OLTs and 33 had stage II OLTs. Group B comprised 29 men and 13 women with a mean age of 34.07 ± 11.84 years and cartilage damage area of 0.4–1.44 cm^2^ (mean, 0.97 ± 0.29 cm^2^). Four patients had MRI stage I OLTs and 38 patients had stage II OLTs. The BMI was calculated according to the World Health Organization classification of BMI (22).

**Figure 1:**
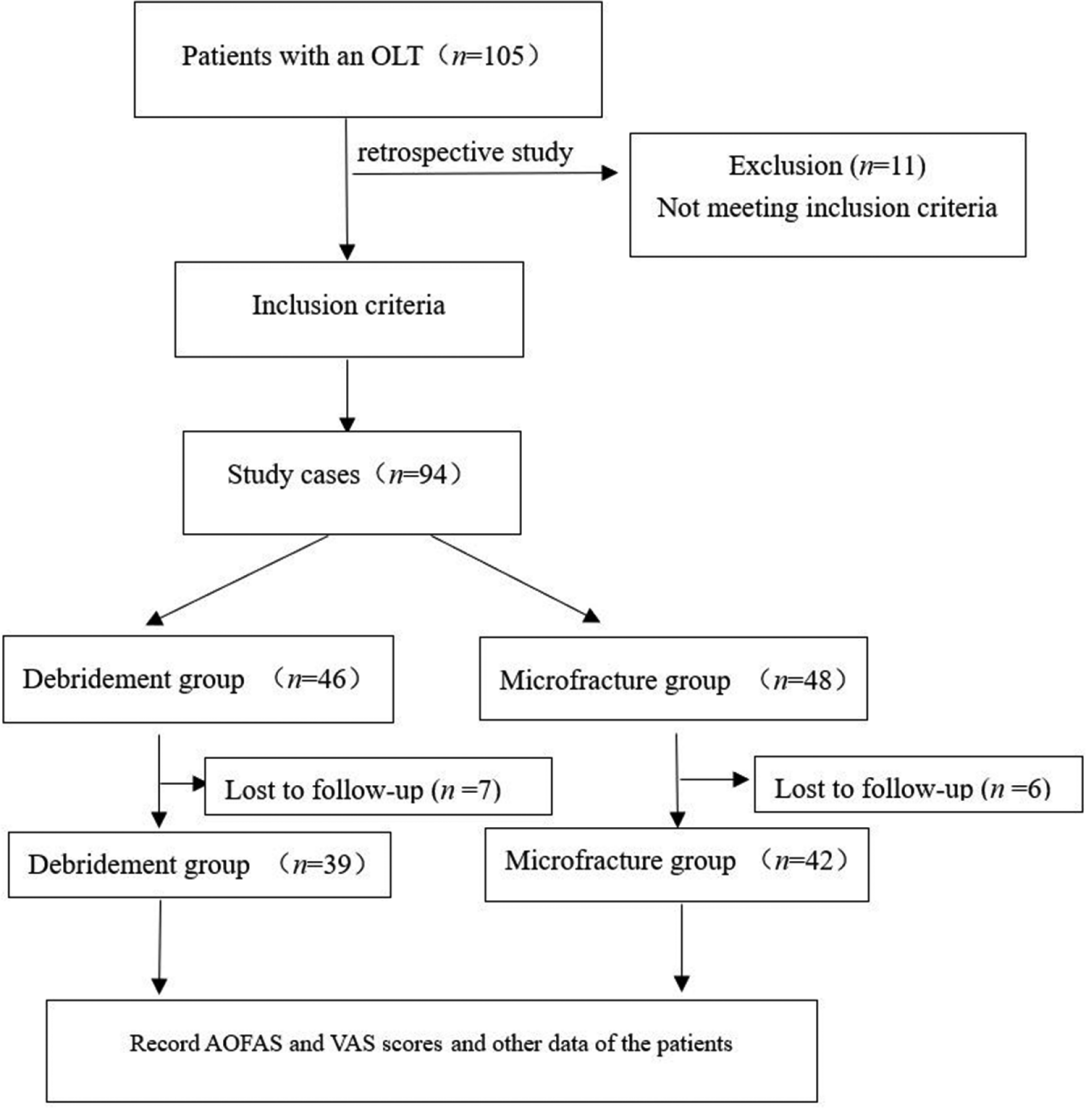
Flow diagram.

This study was performed in compliance with the requirements of the World Medical Association Helsinki Declaration (2013) and was approved by the Medical Ethics Committee of Longyan First Affiliated Hospital of Fujian Medical University (No. 201929). All patients provided written informed consent (Figure 1).

### Preoperative examination

All patients underwent 3.0 T MRI examination before surgery. The maximum length and width of the damaged area were scanned at different levels to calculate the area of osteochondral lesion of the talus. Sequences were obtained in three planes (coronal, sagittal, and axial) by proton density-weighted and fat suppression imaging. Preoperative MRI scans were evaluated by three musculoskeletal radiologists and two orthopedic surgeons, all with more than 10 years of experience (all were experts who independently evaluated the scans). These three musculoskeletal radiologists and two orthopedic surgeons reached a consensus. The scans were evaluated at an image archiving and communication system workstation. All OLTs were consistent with stages I and II. All patients were treated conservatively for 3 months with poor results.

### Surgical technique

All patients underwent spinal anesthesia *via* the lumbar canal and were placed in the supine position. A tourniquet was applied to the affected leg and thigh for hemostasis. A 30-degree, 4-mm-diameter ankle arthroscope (Arthrex, Naples, FL, United States) was used, and either an anterolateral or anteromedial approach was employed. The anterolateral approach was located at the intersection of the external end of the ankle joint line with the third fibular tendon, and the anteromedial approach was located at the intersection of the medial side of the tibialis anterior tendon with the articular line and the lateral side of the saphenous vein and nerves. Arthroscopic examination revealed varying degrees of synovial hyperplasia.

**Figure 2:**
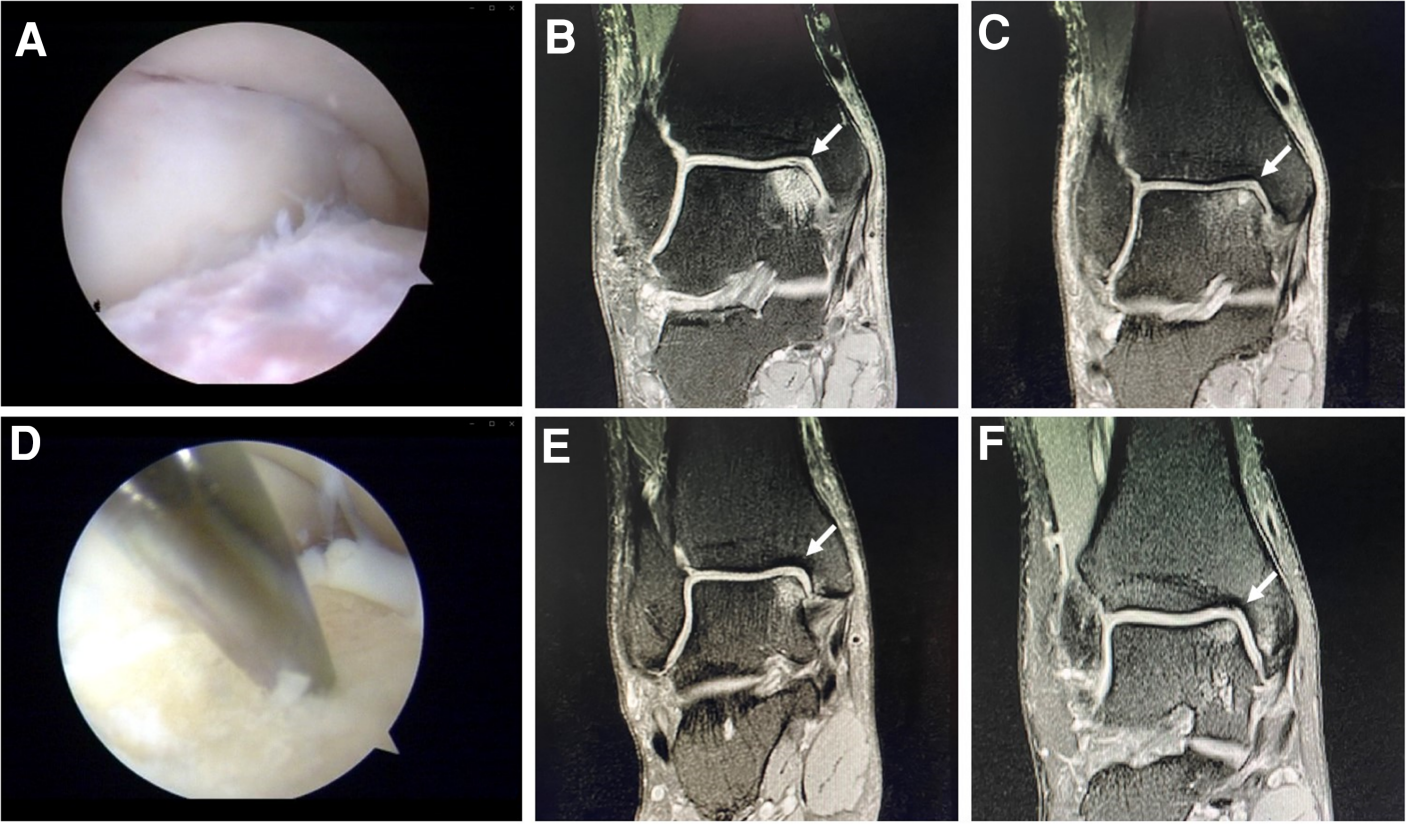
Arthroscopic debridement (**A**). Preoperative (**B**) and postoperative (**C**) MRI of a case in the group A. Microfracture (**D**). Preoperative (**E**) and postoperative (**F**) MRI of a case in the group B.

In Group A, the hyperplastic synovium was removed with curettes and shaver, and the unstable or necrotic cartilage and granulation tissue at the edge of the lesion were cleaned. The subchondral surface was then freshened with shaver and allowed to bleed slightly (23) (Figure 2A).

In Group B, the hyperplastic synovium was removed with curettes and shaver, and the unstable or necrotic cartilage and granulation tissue at the edge of the lesion were cleaned. The microfracture instrument (Arthrex) was used to perform the microfracture operation at the cartilage defect position and the talus subchondral bone plate (depth, 3 mm; spacing, 3–4 mm). If subchondral cysts were present, debridement was performed on the inside of the cysts. No other operations such as bone grafting were performed after microfracture of the capsule wall. The tourniquet was then relaxed; if blood or fat tissue leaked through the hole, the hole was properly perforated. If no blood leakage was observed, the hole was further deepened (24) (Figure 2D). Ligament repair was performed for lateral malleolar ligament injury, and the patients were then treated with a thick cotton pad dressing and posterior plaster splint.

### Postoperative treatment: elevation of affected limb, administration of prophylactic anti-infection treatment, and intermittent dressing changes

In the first 6 weeks after surgery, the patients walked using a crutch, placing no weight on the affected limb. Passive ankle activity was carried out twice a day for 15–20 min each time. At 6–8 weeks after the operation, the patients began partial weight-bearing on the affected limb under the crutch. Passive ankle activity was carried out twice a day for 15–20 min each time. From 8 to 12 weeks after the operation, the patients walked with a full load on the affected limb, and the walking time was gradually extended according to their condition. Two to three times a day, the patients performed 5–10 consecutive 2- to 5-minute static squatting exercises with 30-second rest intervals. Jogging, climbing, and other sports were carried out according to the patient's proprioception and balance training (20, 25). The range of motion and wound recovery were reviewed after 12 months of follow-up.

### Evaluation criteria and observation indicators of curative effect

The patients' degree of pain was scored according to a visual analogue scale (VAS) (26). Joint function was graded using the American Orthopaedic Foot and Ankle Society (AOFAS) score (27). A score of ≥90 was considered excellent, 80–89 was considered good, 70–79 was considered fair, and ≤69 was considered poor. The good/excellent rate (28) was calculated as follows: (number of excellent cases + number of good cases)/total cases × 100%. VAS scores and AOFAS scores were obtained before surgery and at the last follow-up.

### Statistical analysis

SPSS ver. 23.0 statistical software (IBM Corporation, Armonk, NY, United States) was used for the statistical analysis. Measurement data are expressed as mean ± standard deviation; repeated-measures analysis of variance was used for comparison at different time points within a group, and the independent-samples *t* test was used for comparison between groups at the same time points. Count data are expressed as percentage and were analyzed using the *χ*^2^ test. A *P*-value of <0.05 was considered statistically significant.

## Results

**Figure 3:**
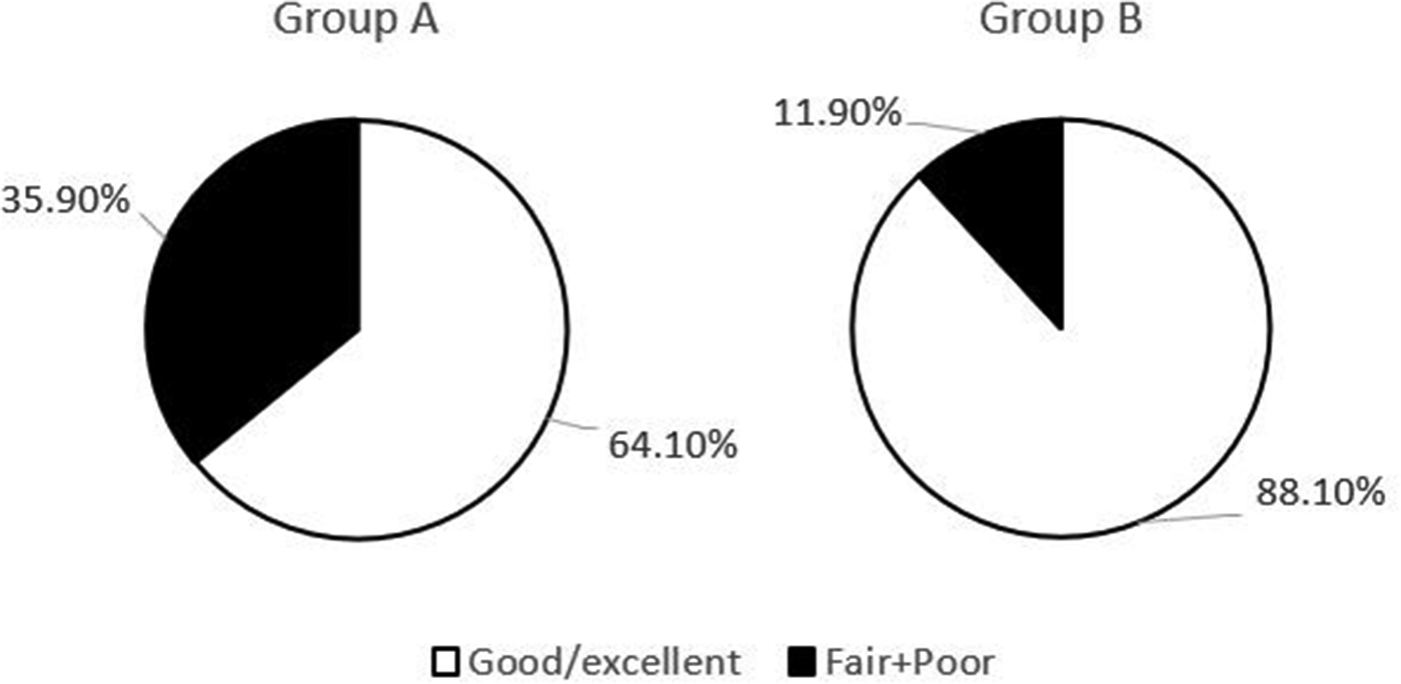
Comparison of good/excellent rates of AOFAS score between the two groups.

The postoperative follow-up duration was 1 year. There was no statistically significant difference in sex, age, disease course, BMI, VAS score, AOFAS score, or cartilage injury area between the two groups (Table 1). The VAS score in Group A decreased from 6.44 to 3.92, and that in Group B decreased from 6.38 to 2.05. The AOFAS score in Group A improved from 40.9 to 82.26, and that in Group B increased from 38.12 to 87.38 (*P* < 0.05) (Table 2). At the last follow-up, the AOFAS score in Group A indicated good/excellent joint function in 25 (30.86%) patients, fair in 10 (12.35%), and poor in 4 (4.94%). The AOFAS score in Group B indicated good/excellent joint function in 37 (45.68%) patients, fair in 4 (4.94%), and poor in 1 (1.23%). There was a statistically significant difference between the two groups (*P *< 0.05) (Table 2). The good/excellent rate in Group B was significantly higher than that in Group A (*P *< 0.05) (Figure 3). MRI at final follow-up showed more obvious cartilage regeneration in Group B than in Group A (Figures 2B,C,E,F). The patients with an overall excellent AOFAS score at the last follow-up comprised 32 (39.51%) men and 9 (11.11%) women aged ≤30 years and 11 (13.58%) men and 10 (12.35%) women aged >30 years, and most of the patients with excellent joint function were young men (*P *< 0.05) (Table 3). There was no statistically significant difference in area between patients with excellent (1.09 ± 0.29) and poor (1.07 ± 0.32) scores (*P* > 0.05). There was also no significant difference in disease course between patients with excellent (225.48 ± 516.13) and fair or poor (168.11 ± 165.83) scores (*P *> 0.05). The BMI of patients with excellent joint function (22.92 ± 2.90 kg/m^2^) was significantly lower than that of patients with fair and poor joint function (25.71 ± 2.12 kg/m^2^) (*P *< 0.05) (Table 4). In Group A, there was a statistically significant difference in the BMI between patients with a good/excellent outcome (23.04 ± 2.66) and those with a fair or poor outcome (25.91 ± 2.11) (*P *< 0.05). In Group B, the BMI was not significantly different between patients with a good/excellent outcome (22.84 ± 3.08) and those with a fair or poor outcome (25.12 ± 2.27) (*P *> 0.05) (Table 5).

**Table 1:** Comparison of preoperative characteristics between the two groups.

Group	Group A	Group B	Test statistics	*P*
M	26 (32.1%)	29 (35.8%)	0.053	0.819
F	13 (16.05%)	13 (16.05%)		
Stage I	6 (7.41%)	4 (4.94%)	0.642	0.51
Stage II	33 (40.74%)	38 (46.91%)		
Age (year)	33.1 ± 11.86	34.07 ± 11.84	−0.376	0.708
BMI	24.07 ± 2.82	23.11 ± 3.07	1.463	0.147
Disease course (day)	219.23 ± 579.10	204.39 ± 314.31	0.145	0.885
VAS	6.44 ± 1.38	6.38 ± 1.65	0.526	0.526
AOFAS	40.90 ± 16.33	38.12 ± 16.78	0.754	0.453
Area (cm^2^)	1.01 ± 0.31	0.97 ± 0.29	0.509	0.612

Data are presented as *n* (%) or mean ± standard deviation.

M, male; F, female; BMI, body mass index; VAS, visual analogue scale; AOFAS, american orthopaedic foot and ankle society.

*P *< 0.05 indicates a statistically significant difference between the groups.

**Table 2:** Comparison of the two groups at the last follow-up.

Group	Good/excellent	Fair	Poor	VAS	AOFAS
Group A	25 (30.86%)	10 (12.35%)	4 (4.94%)	3.92 ± 2.82	82.26 ± 12.54
Group B	37 (45.68%)	4 (4.94%)	1 (1.23%)	2.05 ± 2.07	87.38 ± 9.32
*V*	6.592			6.129	−2.097
*P*	0.037			0.001	0.039

Data are presented as *n* (%) or mean ± standard deviation.

VAS, visual analogue scale; AOFAS, american orthopaedic foot and ankle society.

*P* < 0.05 indicates a statistically significant difference between the groups.

**Table 3:** Male-to-female ratio of patients with good/excellent joint function aged ≤30 and >30 years.

	M	F	Sum
≤30 year	32 (39.51%)	9 (11.11%)	41 (50.62%)
>30 year	11 (13.58%)	10 (12.35%)	21 (25.93%)
*V*	4.305		10.452
*P*	0.038		0.002

Data are presented as *n* (%).

M, male; F, female.

*P* < 0.05 indicates a statistically significant difference between the groups.

**Table 4:** Comparison of cartilage lesion area, BMI, and disease course between patients with good/excellent joint function and those with fair and poor joint function.

	Area (cm^2^)	BMI	Disease course (day)
Good/excellent	1.09 ± 0.29	22.92 ± 2.90	225.48 ± 516.13
Fair + poor	1.07 ± 0.32	25.71 ± 2.12	168.11 ± 165.83
*t*	0.397	3.439	0.866
*P*	0.693	0.000	0.636

Data are presented as mean ± standard deviation.

BMI, body mass index.

*P* < 0.05 indicates a statistically significant difference between the groups.

**Table 5:** Comparison of BMI in subgroups with different functional outcomes.

Group	BMI	*t*	*P*
Group A			
Good/excellent	23.04 ± 2.66	−3.463	0.001
Fair + poor	25.91 ± 2.11		
Group B			
Good/excellent	22.84 ± 3.08	−1.592	0.119
Fair + poor	25.12 ± 2.27		

Data are presented as mean ± standard deviation.

BMI, body mass index.

## Discussion

Among the available surgical management techniques, arthroscopic debridement and microfracture yields a success rate of approximately 85%. Because arthroscopic debridement and microfracture provides rapid recovery with high cost-effectiveness, technical feasibility, a high success rate, and a low incidence rate of complications, this treatment has been widely accepted as a major therapeutic strategy with good functional prognosis (29, 30). Debridement results in freshening of the cartilage surface and enhanced cartilage growth and healing, but the irregularity and depression of the subchondral bone plate after cartilage debridement may create the risk of hyaline cartilage detachment (31). The basic principle of microfracture is penetration of the subchondral bone to induce a repair reaction. Subchondral bone penetration induces the release of serum factors, resulting in the formation of fibrocartilage that covers the wound. After its initial formation, the fibrocartilage must be stabilized to reach a certain mechanical strength (32). The biomechanical properties of fibrocartilage are inferior to those of hyaline cartilage; over time, fibrocartilage decreases in quality and shows inferior wear characteristics. Microfracture is an effective method to relieve clinical symptoms through subchondral decompression (33). Chuckpaiwong et al. (11) reported good functional results of OLTs treated by arthroscopic microfracture in 105 patients with a mean follow-up of 31.6 months. In another study, the patients' ankle function and quality of life were satisfactorily improved after 6.7 years of follow-up after microfracture (34). Therefore, according to these studies, the long-term clinical outcomes after microfracture are just as good as the short- to medium-term outcomes (30). The present study suggests that existing degenerative changes and persistence of fibrochondral deficiency may be related to poor outcomes. Shimozono et al. (35) studied the morphologic changes in the upper subchondral bone on MRI scans 2 years after microfracture and reported worsening clinical outcomes and poor radiographic results over time.

The VAS and AOFAS scores in both groups were significantly improved at the last follow-up compared with those before surgery, indicating that both debridement and microfracture were effective. The good/excellent rate in Group B was higher than that in Group A (*P* < 0.05), indicating that the effect of microfracture was better than that of debridement. These findings suggest that broken cartilage itself can form cartilage, and microfracture can induce an overflow of growth factors into the bone marrow and promote cartilage growth. Microfracture was also more effective in men aged ≤30 than >30 years. Studies have shown that broken cartilage itself has a high potential to form cartilage (36). Therefore, microfractures promote growth factor exudation and promote cartilage repair. Considering that young chondrocytes have superior chondrogenesis potential, that joint surface fibrillary formation and cartilage degeneration are age-related processes, and that tensile stiffness and strength gradually decline (37), patients are not advised to continue to engage in competitive sports activities or heavy physical labor after surgical rehabilitation. In the present study, the treatment effect in both groups was better in young patients. Various changes in the synthetic properties of articular cartilage as well as increased apoptosis have been shown to weaken the ability of chondrocytes to repair damaged tissues over time (38), and young cartilage produces more proteoglycan C, type II collagen, and IX mRNA than old cartilage. Additionally, the growth of young cartilage cells in monolayer culture is significantly faster than that of old cartilage cells (39). This is considered to be related to the strong ability of chondrocytes to repair damaged tissues, which is consistent with the results of this study.

The reported sensitivity and specificity of MRI for osteochondral lesion of the talus is 96% (40). Surgical treatment is recommended for patients with stage I and II MRI manifestations who undergo failed standard conservative treatment (41). In this study, the stripped talus cartilage and surrounding proliferative tissues were completely debrided by a shaver under arthroscopy (42). Patients in Group B underwent microfracture of cartilage on the basis of complete dissection of proliferating cartilage and peripheral tissue, uneven fibular cartilage in the postoperative cartilage-injured area, excellent midterm follow-up results, and reliable results. Effective repair of talus cartilage lesions and restoration of normal joint function were achieved (12). There was no statistically significant difference in the cartilage damage area or disease course between patients with good/excellent joint function and those with fair and poor joint function. Additionally, there was no significant difference in the cartilage damage area of the patients included in this study (all areas were within 1.5 cm^2^). The patients did not exercise aggressively after surgery, and pain and swelling did not aggravate their condition; thus, there was no difference in the disease course. The BMI of patients with good/excellent joints was significantly lower than that of patients with fair and poor joints, and our analysis was based on the following two reasons: Firstly, obesity has negative physiologic and psychological impacts on patients' postoperative quality of life and affects their postoperative recovery. Secondly, obese patients have a poor prognosis for the development of degenerative joint disease, consistent with previous studies (43, 44). Finally, obesity-associated behavior such as functional limitations, constant dieting, and mental stress from their body image and poor self-esteem, coupled with social stigma, all play a strong role linking obesity with depression (45). In Group B, we concluded that an elevated BMI did not adversely affect pain and function, and a high proportion of patients reported greater postoperative satisfaction and achievement, similar to other studies (44). Instead, patients should be encouraged to normalize their BMI in view of the negative impact on their quality of life and the physical limitations associated with a raised BMI.

At the 2018 International Consensus Meeting on Cartilage Repair of the Ankle (46), complete weight bearing was not recommended within 2–3 months after ankle cartilage surgery. In this study, the patients performed partial weight bearing under a crutch within 2 months after surgery. As their rehabilitation training progressed, the patients could carry out complete weight bearing 3 months after surgery depending on their condition, and jogging, climbing, and other sports activities could be carried out according to their proprioception and balance training. Thus, we believe that the herein-described rehabilitation program is reasonable.

There were still some limitations for the present study. Firstly, the follow-up time was relatively short. Secondly, there was a selection bias in the retrospective study in spite of no significant difference of the basic parameters between the two groups. Finally, not all the patients underwent MRI at final follow-up and most of the results were the subjective evaluation. Further prospective study with more cases, long-term follow-up and objective evaluation was needed in the future.

In conclusion, the microfracture for the treatment of osteochondral lesion of talus is more effective than debridement in improving ankle function, especially in relatively young men with a relatively low BMI.

## Data Availability

The original contributions presented in the study are included in the article/Supplementary Material, further inquiries can be directed to the corresponding author.
